# Double-Unit Superomedio-Central (DUS) Pedicle Inverted-T Reduction Mammaplasty in Gigantomastia: A 7-year Single-Center Retrospective Study

**DOI:** 10.1007/s00266-021-02351-y

**Published:** 2021-06-18

**Authors:** A. Wolter, S. Fertsch, B. Munder, P. Stambera, T. Schulz, M. Hagouan, D. Janku, K. Staemmler, L. Grueter, N. Abu-Abdallah, K. Becker, B. Aufmesser, J. Kornetka, C. Andree

**Affiliations:** 1 Department of Plastic and Aesthetic Surgery, Interdisciplinary Breast Center, Sana Kliniken Duesseldorf GmbH, Graeulinger Strasse 120, 40625 Duesseldorf, Germany; 2 Faculty of Health, University of Witten-Herdecke (UWH), Alfred-Herrhausen-Strasse 50, 58448 Witten, Germany

**Keywords:** Gigantomastia, Macromastia, Severe mammahypertrophy, Reduction mammaplasty, Breast reduction, Superomedial pedicle

## Abstract

**Introduction:**

Reduction mammaplasty in patients with gigantomastia is challenging. The Double-Unit technique with a Superomedio-Central pedicle and inverted-*T* incision is the standard technique for reduction mammaplasty in our clinic. The aim of this study was to review our approach in cases with gigantomastia in comparison with the current literature.

**Patients and Methods:**

From 01/2011 to 12/2017, we performed 831 reduction mammaplasties in 630 patients. The *Double-Unit*  *Superomedio-Central*  *(DUS) pedicle* and inverted-*T* incision was implemented as a standard procedure for gigantomastia. Patient demographics and the outcome parameters complication rate, patient satisfaction with the aesthetic result, nipple sensibility, and surgical revision rate were obtained and retrospectively analyzed.

**Results:**

In 37 patients, 55 reduction mammaplasties were performed with more than 1000 g per breast. Mean resection weight was 1311 g on right side and 1289 g on left side. Mean age was 52.5 years, mean body mass index was 32.8 kg/m^2^, mean sternal-notch-to-nipple distance was 38.3 cm. A free NAC graft was necessary in four breasts. Overall complication rate was 14.5%; secondary surgical revision rate was 12.7%. 91% of the patients were “very satisfied” and “satisfied” with the aesthetic result. Nipple sensibility was rated “high” and “medium” in 83%.

**Conclusion:**

The *Double-Unit* technique with a *Superomedio-Central *pedicle and inverted-*T* incision is very effective to achieve volume reduction and aesthetically pleasing reproducible results with a low complication rate in cases with gigantomastia.

**Level of Evidence:**

*Level of Evidence * This journal requires that authors assign a level of evidence to each article. For a full description of these Evidence-Based Medicine ratings, please refer to the Table of Contents or the online Instructions to Authors www.springer.com/00266.

**Supplementary Information:**

The online version contains supplementary material available at 10.1007/s00266-021-02351-y.

## Introduction

“*Gigantomastia*” describes a rare extreme hypertrophy of the female breast. Although there is no universally accepted definition, many authors cite gigantomastia as breast enlargement that requires a reduction mammaplasty of >1000 g per breast [1–4]. Various procedures have been described for reduction mammaplasty with specific skin incisions, patterns of breast parenchymal resection and retained blood supply to the remaining breast tissue and nipple–areolar complex (NAC) [5, 6]. To date, only a limited number of publications can be found in literature addressing the challenging condition of gigantomastia [2–4, 7–12].

A very important issue in breast reduction surgery is the preservation of the vascularity of tissues as well as sensibility, especially of the NAC. Various pedicle techniques have been described in breast reduction surgery [13]. The superomedial pedicle is commonly used in Europe [9, 14], whereas the inferior or central pedicle is favored in the USA [2]. In case of an extremely elongated sternal-notch–NAC (SN-NAC) distance of > 40 cm or more, many authors recommend a free NAC grafting [15, 16]. The superomedial dermal pedicle for NAC transposition was first described by Orlando and Guthrie for use in reduction mammaplasty and mastopexy [17].

Elizabeth Hall-Findlay described in 1999 a vertical scar medial (or superomedial) pedicled breast reduction technique as a modification to the standard Lejour [18] vertical reduction mammaplasty, and this technique has grown rapidly in popularity [19–21]. There is no difficulty in insetting the NAC to its new site, and this technique is safer than the superior pedicle vertical technique in terms of NAC circulation. Some modifications of the “Hall-Findlay Technique” have already been published [22–24] but none to date addressing gigantomastia cases. However, the Hall-Findlay technique is not without problems, like bottoming out and dog ears in the inframammary fold (IMF). In her book, E. Hall-Findlay describes in 2011 the necessity of an inverted-*T* scar regarding two patient examples: one case after massive weight loss and one with gigantomastia [25]. Especially in gigantomastia cases, NAC ischemia is a threatening complication. The two novelties presented in our study are an horizontal incision at the IMF and the preservation of the fibrous horizontal septum (described by Wueringer et al. [26]) with its containing vessels included in the central pedicle part additionally to the superomedial pedicle (*Double-Unit Superomedio-Central *(*DUS*)* Pedicle*). To date, there is no relevant publication addressing this technique in gigantomastia cases. The purpose of this study is to show a modification of Hall-Findlay’s technique with a Double-Unit Superomedio-Central pedicled inverted-*T*-scar reduction mammaplasty in gigantomastia and a retrospective analysis regarding complications, patient satisfaction, and NAC sensibility.

## Patients and Method

The records of 630 patients who underwent 831 reduction mammaplasties under general anesthesia from January 2011 to December 2017 were retrospectively reviewed. A resection weight of more than 1000 g per breast corresponding to the definition of gigantomastia was defined as inclusion criterion. The data collected included patient demographics (age, body mass index (BMI kg/m^2^), SN-NAC distance, operation time, hospital stay, and amount of resected breast tissue (Table 1). Outcome parameters such as complication rate, patient satisfaction with the aesthetic result, nipple sensibility, and the secondary revision rate were recorded and evaluated (Tables 2 and 3). All patients were photographed preoperatively, 6 months, and 1 up to 4 years postoperatively in standard perspectives.

**Table 1:** Patient demographics (36 patients, breasts: *n* = 55)

Overall collective	
Patients	*n* = 36, 55 breast reductions
Age (years)	52.5 (18–76)
BMI (kg/m^2^)	32.8 (22–40.5)
Sternal notch–NAC distance (cm)	38.3 (27–56)
Operation time (min)	164 (76–288)
Hospital stay (days)	4.9 (2–9)
Resection weight right side (g)	1311 (1000–4200)
Resection weight left side (g)	1289 (1005–4600)
Free NAC grafting	4 breasts (7.2%)

(*BMI* body mass index kg/m^2^, *NAC* nipple–areolar complex)

**Table 2:** Outcome parameters—Complications and surgical revisions

Outcome parameter	Overall collective *n* = 55 breast reductions
**Overall** **Complications**	8 (14.5%)
***Minor***	6 (10.9%)
NAC epidermiolysis	2 (3.6%)
Seroma	1 (1.8%)
Local wound infection	1 (1.8%)
Delayed wound healing (*T* point)	2 (3.6%)
***Major***	2 (3.6%)
Total NAC necrosis	1 (1.8%)
Acute hematoma with revision	1 (1.8%)
**Secondary revisions**	7 (12.7%)
Contour revisions	3 (5.5%)
Scar revisions	2 (3.6%)
NAC revisions	2 (3.6%)

(*NAC* nipple–areolar complex)

**Table 3:** Outcome parameters—Patient survey evaluation

	Patient survey evaluation *n* = 33/36 (92%) patients
**Patient satisfaction**	
1 = Very satisfied	21 (64%)
2 = Satisfied	9 (27%)
3 = Less satisfied	2 (6%)
4 = Not satisfied	1 (3%)
**NAC sensibility**	*n* = 42 (free NAC grafts excluded)
1 = High	21 (50%)
2 = Medium	14 (33%)
3 = Low	4 (10%)
4 = No sensation	3 (7%)

(*NAC* nipple–areolar complex)

## Markings

**Fig. 1:**
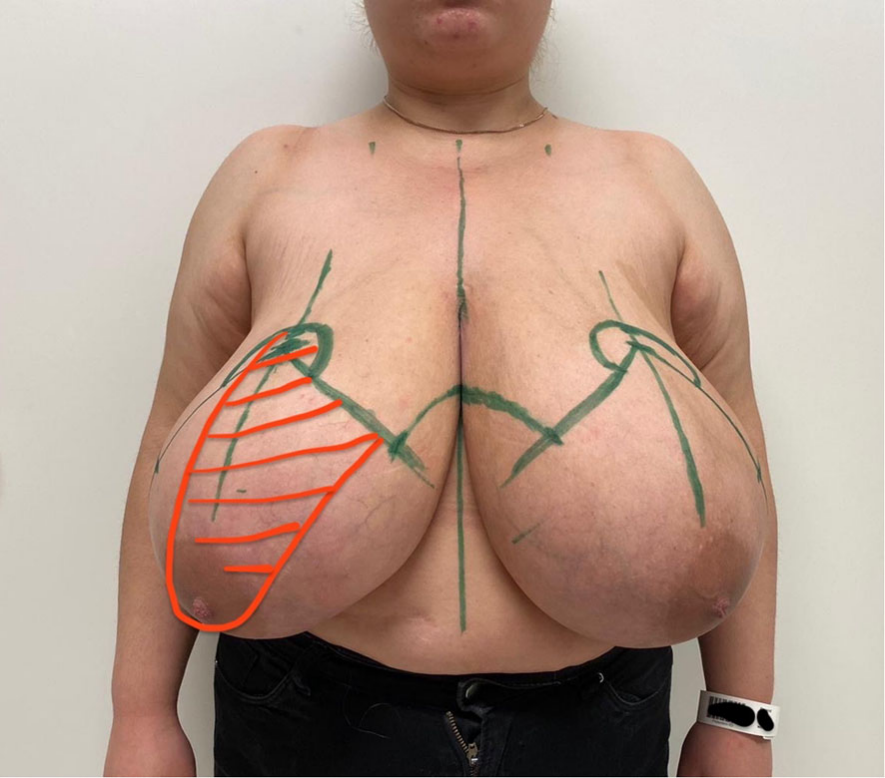
Patient example with ***Pedicle Marking*** (*red color*) of the *Double-Unit Superomedio-Central* (*DUS*) *Pedicled* Inverted-T Reduction Mammaplasty (*green color*)

**Fig. 2:**
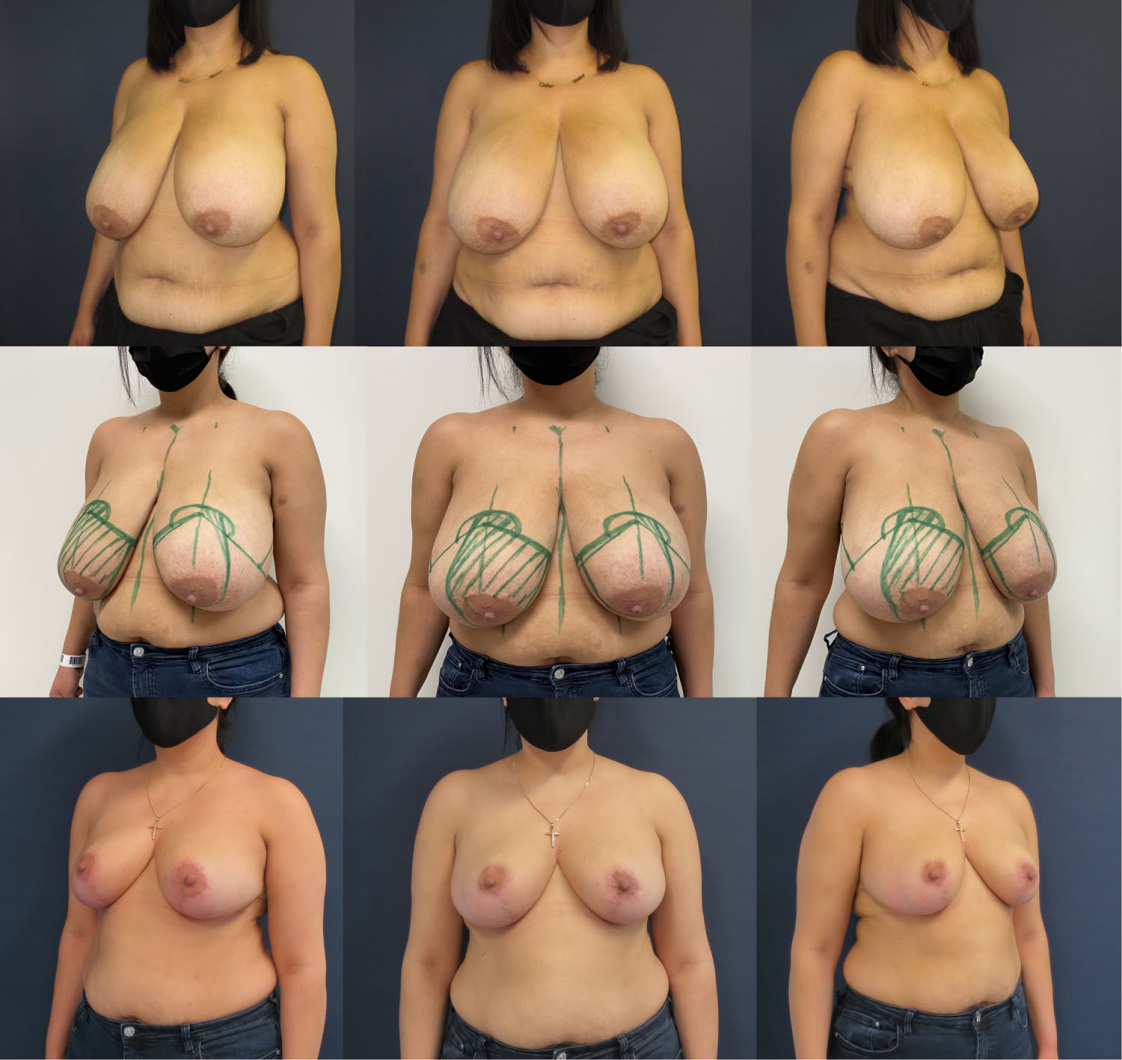
**Patient example 1** with *DUS-Pedicle marking*. 37-year-old patient with cup size 90 G/H, SN-NAC distance 35 cm right side and 34 cm left side, ptosis grade III by Regnault, BMI 30.5 kg/m^2^. Preoperative status (*above*), preoperative markings (*middle*), and 12 months postoperative (*below*) after *Double-Unit Superomedio-Central (DUS) Pedicled *Inverted-*T* Reduction Mammaplasty, form stable breast shape and good upper pole projection. Resection weight *right side* **1309 g** and *left side* **1185 g** (BMI: body mass index kg/m^2^; SN: sternal notch, NAC: sternal notch–nipple–areolar complex)

**Fig. 3:**
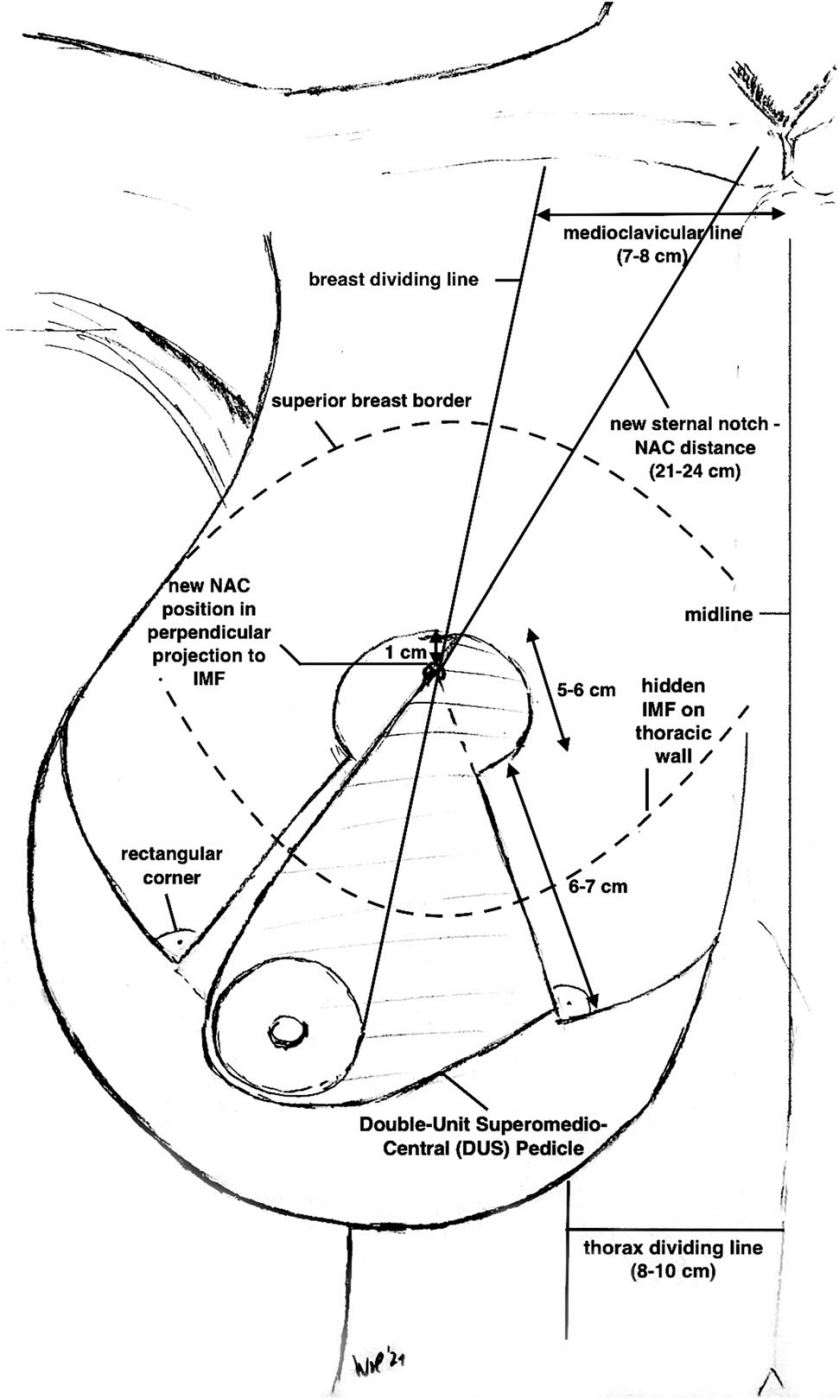
Illustration of ***Surgical Markings*** and measurements of the *Double-Unit Superomedio-Central* (*DUS*) *Pedicle* in a schematic gigantomastic breast (NAC: Nipple–Areola Complex; IMF: Inframammary Fold)

Preoperative markings were made while the patient was standing according to the standard reduction mammaplasty in our clinic (Figs.1, 2 and 3 and Video File 1).

## Surgical Technique

**Fig. 4:**
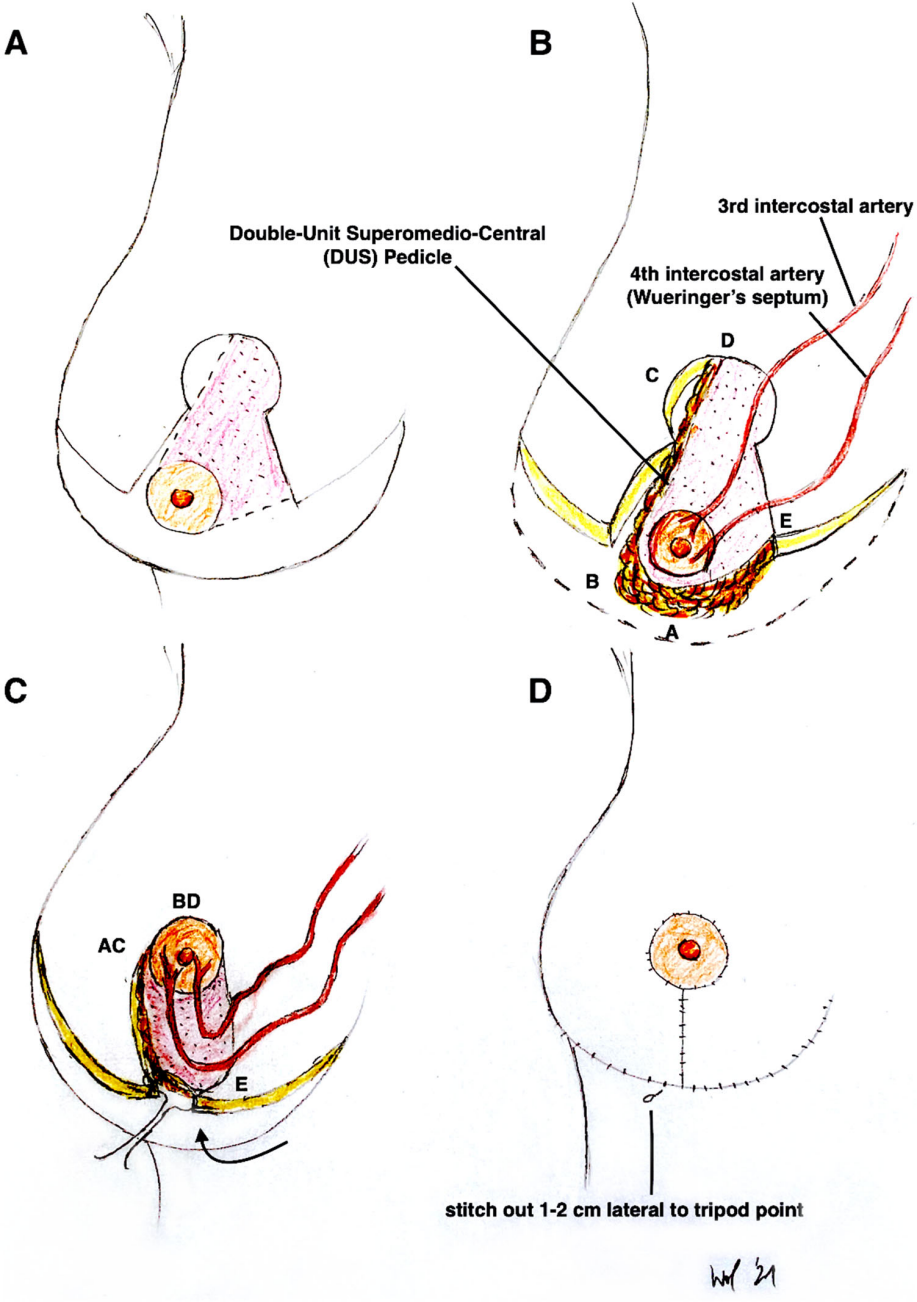
Illustration of **Surgical Technique**. **A**, **B** *Double-Unit Superomedio-Central *(*DUS*)* Pedicle* Inverted-*T* Reduction Mammaplasty with illustration of vascular supply. **C** Cranial rotation of *DUS-Pedicle* and inset of NAC in new position. **D** Skin closure and stitch-out laterally to vulnerable *tripod zone*. (NAC: nipple–areola complex)

**Fig. 5:**
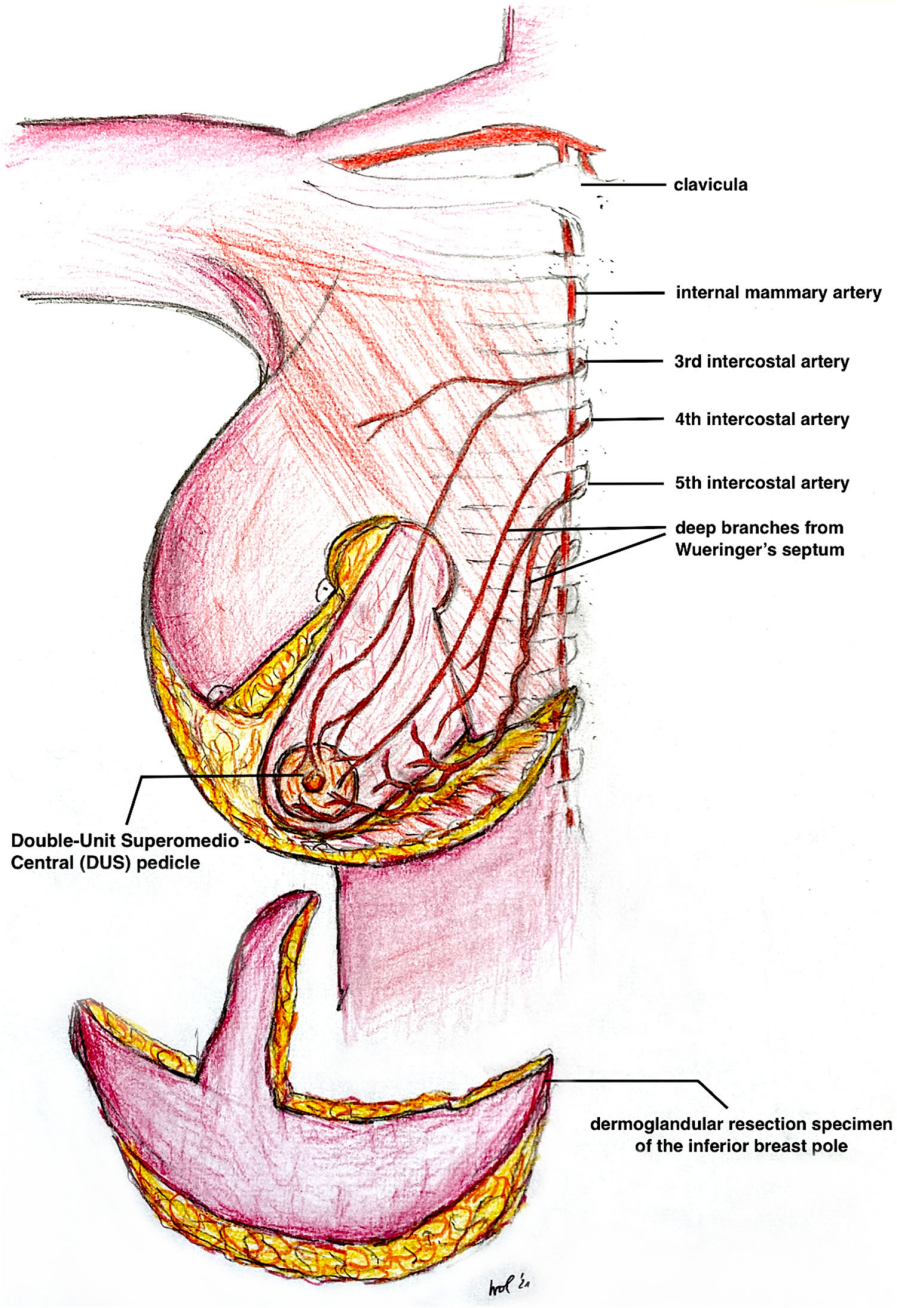
**Anatomical Illustration** of the *Double-Unit Superomedio-Central *(*DUS*)* Pedicle,* the vascular supply of the NAC and the *Wueringer’s* horizontal septum [26] in ***frontal*** view. (NAC: nipple–areola complex)

**Fig. 6:**
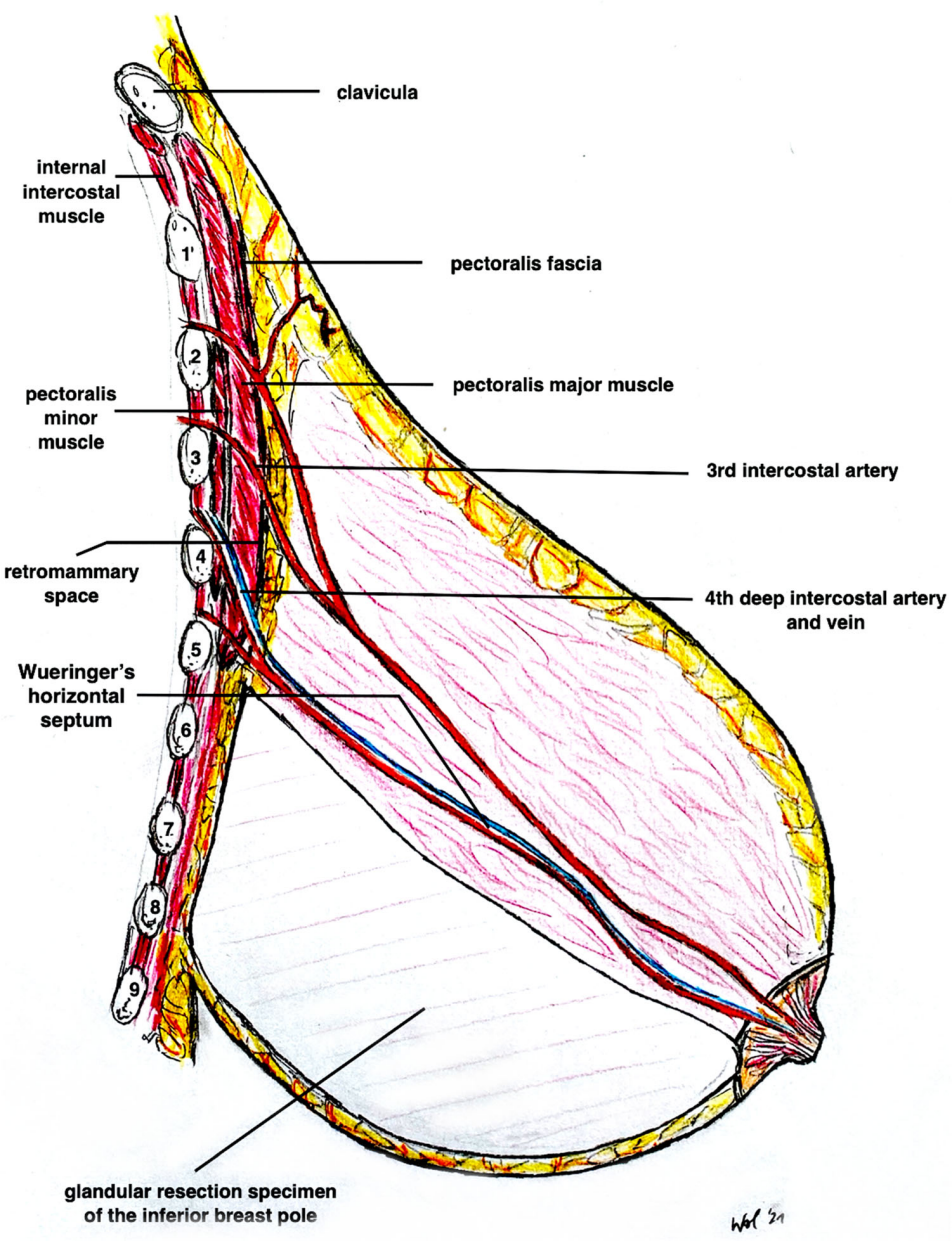
**Anatomical illustration** of the *Double-Unit Superomedio-Central *(*DUS*)* Pedicle,* the vascular supply of the NAC and the Wueringer’s horizontal septum [26] in ***sagittal*** view. (NAC: nipple–areola complex)

Surgery was performed under general anesthesia with the patient in a supine position (see also Fig. 4 and Video File 2). After a single-shot antibiosis, the NAC was marked with a “cookie cutter” (38–42 mm). We used a temporary tourniquet of the breast to facilitate the deepithelialisation process. The NAC-bearing pedicle was then de-epithelialized with scissors or scalpel with special regard to the preservation of the subdermal venous plexus. The Superomedio-Central pedicle was prepared down to the pectoralis fascia under preservation of the fibrous horizontal septum, described by Wueringer et al. [26]. The horizontal septum is a thin layer of connective tissue that arises from the pectoralis fascia at the level of the fifth rib and reaches the NAC. It divides the breast into cranial and caudal parts (Figs. 5, 6 and Figs. 4A + B). In gigantomastia, the vascular anatomy of the breast remains but the breast is more ptotic with an increased SN-NAC distance and broad base. The vascular supply to NAC relies mainly on perforating arterial branches from the internal mammary artery, the lateral thoracic artery at the level of the 2nd and 3rd intercostal artery, and the anterior intercostal artery at the level of the mid fourth and mid fifth intercostal space (Figs. 5, 6 and Fig. 4B). The fibrous horizontal septum includes the perforators from the anterior intercostal artery emerging from the pectoralis major muscle at the level of the fourth and fifth intercostal space. Our technique combines the superomedial pedicle and the central horizontal Wueringer’s septal branches (*Double-Unit Superomedio-Central *(*DUS*)* Pedicle*).

Undermining of the pedicle should be avoided to preserve the vascularity and nerve insertions to ensure a *Double-Unit* NAC-bearing pedicle consisting of a *superomedial* and *central* part. Surgical “en bloc” excision of skin, fat and gland around the pedicle with a *C*-shaped pattern (as described by Hall-Findlay [20]) was performed as outlined by skin markings (Figs. 1, 2 and 3). The resection was stopped when the inferior border of the pectoralis major muscle fascia was reached. Resection was then continued to the lateral extension and inferomedial portion of the breast; in some cases with a moderate trimming in the lateral portion of the new NAC area and in individual cases to decrease the volume of a bulky lateral breast pillar. The NAC was then tension-free rotated for inset to its new position and temporarily stapled (Fig. 4C). Symmetry was then checked at the upright sitting patient. After elevation of blood pressure by the anesthesiologists to a systolic minimum of 130 mmHg, we irrigated the wounds with warm saline solution and performed a meticulous coagulation. A 12-mm single drain was placed for each breast laterally. Closure was commenced laterally to take up the excess skin and reduce tension at the *T* junction. The medial and lateral breast pillars were sutured in two planes, respectively deep in the parenchyma with 2-0 PDS single knots to avoid a bottoming out and to preserve upper pole projection. The anchor suture in the *T*-junction point was performed with a resorbable 2-0 Monocryl single knot. Skin closure was performed with resorbable 3-0 Monocryl single knots and a running suture intracutaneously in the IMF and vertically. The NAC’s skin closure was performed with a 4-0 resorbable Monocryl double running suture intracutaneously (Fig. 4D).

Only in cases with a sternal-notch–NAC distance > 45 cm, we adopted the free NAC graft technique and used a pseudo-pedicle prepared as described before as NAC recipient site. In all cases, the resected breast glandular tissue was analysed histopathologically. Drains were removed when secretion decreased below 30 ml in 24 hours. Early mobilization and anticoagulation with low-dose heparin were prescribed directly after surgery. Wound dressing was completed by a right fitting compression bra applied immediately in the operating room. All patients were advised to wear this compression bra and to avoid excessive sport and exercises for at least 6 weeks.

To evaluate our results qualitatively, we recorded the complication and secondary revision rate (Table 2). Moreover, a patient satisfaction survey concerning the aesthetic result and a subjective assessment of nipple sensibility were performed as follows (Table 3). Patient satisfaction could be rated as “very satisfied” (1), “satisfied” (2), “less satisfied” (3) and “not satisfied” (4). Nipple sensibility was subjectively evaluated per breast side as “high” (1; 1st degree), “medium” (2; 2nd degree), “low” (3; 3rd degree) and “no sensation” (4; 4th degree).

Patients presented to our outpatient department for follow-up at 2 weeks, 6 months, 1 year up to 4 years after surgery or were questioned by telephone interview. The collected data were then transferred into Excel.

In our institution we have a very constant team of six operating plastic surgeons (head of department, five attendings, all German Board, one additionally European Board (EBOPRAS) certified, and six residents) to ensure uniformity of technique and follow-up observation. The surgical team usually consists of one or two attendings and one resident.

## Results

From January 2009 to December 2017, we performed 831 reduction mammaplasties in 630 patients. Fifty-five reduction mammaplasties (7%) fulfilled the inclusion criteria for gigantomastia with a resection weight of more than 1000 g per breast. The mean age was 52.5 years (range: 18–76 years), mean distance between sternal notch and NAC was 38.3 cm (range: 27–56 cm), mean body mass index (BMI kg/m^2^) was 32.8 kg/m^2^ (range: 22–40.5 kg/m^2^),). The mean operating time lasted 164 min (range: 76–288 minutes). The mean weight of resected tissue was 1311 g (range: 1000–4200 g) on the right side and 1289 g (range: 1005–4600 g) on the left side. The mean hospital stay was 4.9 days (range: 2–9 days).

Overall complication was 14.5% (Table 2). Complications were divided into minor, which could be managed conservatively and major complications, where surgical revision was necessary. Minor complications included seroma, NAC epidermiolysis, local wound infection and delayed wound healing in the *T*-junction. Major complications included total NAC necrosis and acute hematoma with revision.

There was only one case with an acute hematoma requiring evacuation (1.8%) and one full NAC necrosis (1.8%) that was reconstructed by skate flap and areolar tattooing. As minor complications there were two NAC epidermiolysises (3.6%) that healed by secondary intention, one seroma (1.8%) that was evacuated by needle aspiration, one local wound infection (1.8%) treated by local antiseptic ointment and antibiotics and two delayed wound healings in the *T*-junction (3.6%), that healed completely by secondary intention. Secondary surgical revisions for aesthetic improvement (e.g., contour revisions, scar revisions or NAC revisions) were necessary in seven cases (12.7%). Flap/pedicle or steatonecrosis did not occur. 

Thirty-three patients (92%) could be interviewed during the follow-up appointments and by telephone survey. The mean follow-up time was 24 months (12–48 months). The patient survey revealed a high satisfaction rate with the aesthetic result (Table 3). 21 patients (64%) rated the results as “very satisfied” (1), 9 patients (35%) as “satisfied” (2), 2 patients (6%) were “less satisfied” (3), one patient (3%) was „not satisfied“ (4). Nipple sensibility was rated subjectively by the patients in 21 NACs (50%) as “high” (1) and in 14 NACs (33%) as “medium” (2), in 4 NACs (10%) as „low“ (3) and in three NACs (7%) with „no sensation“ (4) (one total NAC necrosis). Cases with free NAC grafting were excluded in the sensibility analysis. No malignant or pathological findings were seen in the histopathological analysis.

## Discussion

Over the last decades, breast reduction techniques became numerous. Gigantomastia breast reduction, in particular, is still challenging and has a high complication rate. Factors that can negatively affect the outcome of a reduction mammaplasty have already been previously frequently described (e.g., age, BMI kg/m^2^, grade of ptosis, comorbidities, smoking and amount of resection weight) [27, 28]. Due to fact that NAC necrosis and loss of NAC sensation are the most severe complications of reduction mammaplasty, safety is mainly dependent on assuring blood and nerve supply to the NAC. The complication of NAC necrosis in breast reduction and mastopexy has been reported up to 7.3% [8]. Due to the severely increased SN-NAC distance in gigantomastic breasts, the vascular safety of the NAC remains a primary concern. In normal-sized breasts reduction mammaplasties the inferior, superior, medial or lateral pedicle provides adequate blood supply to the NAC, but might not include sufficient arterial flow and venous output to the NAC in cases of gigantomastia. Van Deventer et al. analysed the arterial breast blood supply through a cadaver research project and concluded that even though the main sources are constant (internal/lateral thoracic, anterior intercostal and acromiothoracic artery), partial or complete absence of branches can occur. Due to this unpredictable anatomy and blood supply of the NAC and to reduce the risk of potential NAC loss, they recommended to use a technique including branches from more than one source [29]. Palmer and Taylor analysed the vascular territories of the breast and found the internal thoracic artery to be the dominant blood supply in 70 percent of patients [30]. Furthermore, the only vessel to contribute at least one perforator to the NAC in 100 percent of cases was the internal mammary artery. The superomedial pedicle (which includes these perforators) is therefore a sound anatomical choice. First described by Orlando and Guthrie [17], the superomedial pedicle technique has been demonstrated to be both safe and reliable [3].

**Fig. 7:**
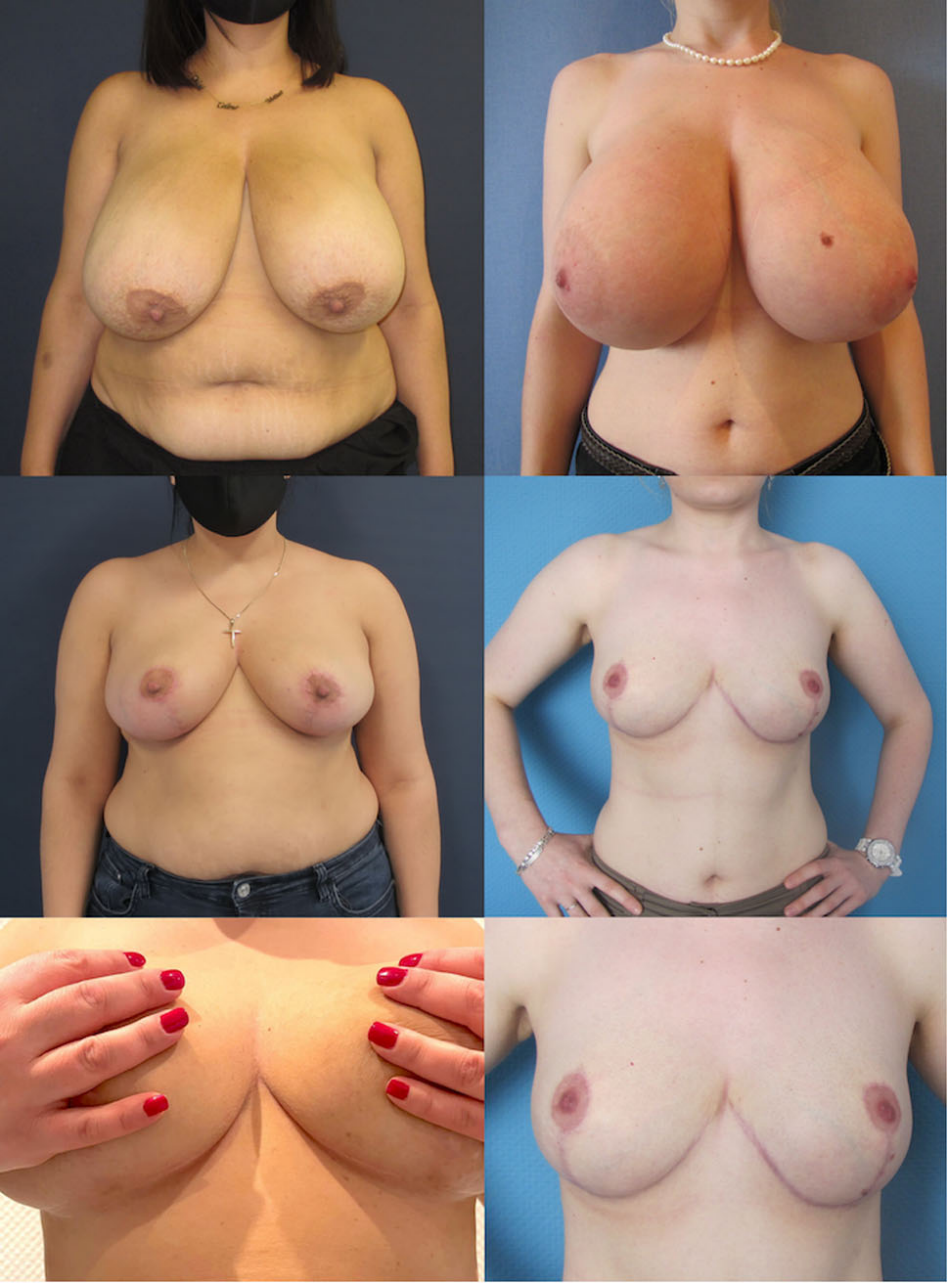
Patient examples (left side: patient Fig. 2 and right side: Fig. 10) pre- and postoperative with focus on the **inframammary (IMF) scar**; note: very slight *bottoming out* in the left case due to *advanced age* (37 years (*left side*) vs. 23 years (*right side)*), *impaired skin quality* and *elasticity,* more *severe* grade of *breast ptosis* and status after *breast feeding*

In the last decade, the superomedial pedicle with vertical reduction has gained popularity, particularly Elizabeth Hall-Findlay’s medial (or superomedial) pedicle vertical reduction mammaplasty technique [4, 20]. On the other hand, it is not easy to achieve perfect results in gigantomastia cases who have undergone vertical scar breast reduction techniques [31]. Thus, in these cases, the superomedial and inferior pedicle with Wise pattern skin excision is preferred by many authors [9]. Le Roux et al. published an anatomical study concerning the preservation of essential venous drainage networks in breast surgery and claimed the superomedial/medial and inferior pedicles to contain the most extensive venous drainage patterns [24]. Reduction mammaplasty with the inferior pedicle is a well-established technique and applicable in a wide range of breast sizes and the complication rate is rather low [32]. Although this is a reliable option for larger resections, development of “bottoming out” is a major criticism of this technique. Especially in gigantomastia cases with extensively impaired skin quality and elasticity, sagging of breast tissue below the inframammary scar is a potential problem. Although the majority of our patients were very satisfied with their outcomes and none complained of a ‘‘bottoming-out’’ deformity, this issue was slightly noticeable in very few patients in our collective (see also patient examples regarding the IMF scar Fig. 7). To avoid this phenomenon even in very massive cases, due to the gravity forces of tissue in the lower pole and the severely impaired skin quality, the NAC should not be placed too high (optimally in perpendicular projection to the IMF) and the vertical pillar limbs should not be planned too short or too long (see also “Markings” Fig. 3).

The superomedial pedicle on the other hand is potentially versatile and can be used with various skin reduction techniques. Furthermore the reliability and safety of the superomedial pedicle have been frequently reported [4, 23, 33]. Compared with the inferior pedicle, the superomedial pedicle causes increased breast projection and saves operating time in vertical scar reductions [34].

To modify the Hall-Findlay technique with medial (or superomedial) pedicle, we decided to combine the superomedial pedicle with the central glandular part (including the intercostal perforators from the internal mammary artery and vein). The central pedicle technique was first described by Hester al. in 1985 [35] and later by Wueringer [36]. Therefore, by combining the superomedial and central pedicle with preservation of Wueringer’s septum in this “*Double-Unit Superomedio-Central (DUS) Pedicle*” technique the risk of NAC loss can potentially be reduced including reliable arterial sources and preserving a sufficient venous NAC drainage. A similar principle was recommended by Bucaria et al. in severe breast ptosis cases [37]. The authors could also show a very low complication rate, especially concerning the risk of a complete NAC loss. The preservation of the fourth and fifth anterior intercostal perforators enhances the vascularization of the breast parenchyma and NAC [11, 26]. Their preservation can be obtained by avoiding any dissection over the pectoralis major muscle, so that Wueringer’s [26] septum is preserved. Thus this modified technique can be regarded as a combination of the central mound and the superomedial pedicle techniques (Figs. 1, 2, 3, 4, 5, 6). The broad dermoglandular pedicle together with the oblique design allows for a safe arc of rotation, preventing kinking of the pedicle. In addition to being safe and reliable, the technique has been shown to be relatively quick to perform, saving operating time (mean operation time: 164 min).

In the past, the focus has also placed on reducing the length of the scars, respectively by *J* or *L* scars [32, 38], ending with vertical scar mammaplasty like in Lejour’s or Hall-Findlay’s technique [10, 18, 20, 21]. Dog-ear deformity at the IMF is always a potential problem in vertical mammaplasty techniques. Expecting that part to settle down in a few months is sometimes in vain. Some authors suggest to place a purse-string suture below the fold [22], but tension can cause wound deshiscences in some cases. In our opinion, patients usually not complain about the inframammary scar as long it is located within the IMF and maximal breast projection area. Furthermore, the shape of the reduced breast should not be compromised to shorten the scars. In gigantomastia, large volumes are usually associated with ptosis, axillary extensions, and hollowness of the upper breast pole. To avoid potential scar, dog ear or volume revisions in the IMF or in the lateral part of the breast, we adopted a wise pattern inverted-*T* incision. The inframammary scar only exceeded the width of the reduced breast in case of avoiding dog ears in severe lateral bra rolls and remained “hidden” in the IMF or its extension. The length of this scar was reduced whenever possible.

**Fig. 8:**
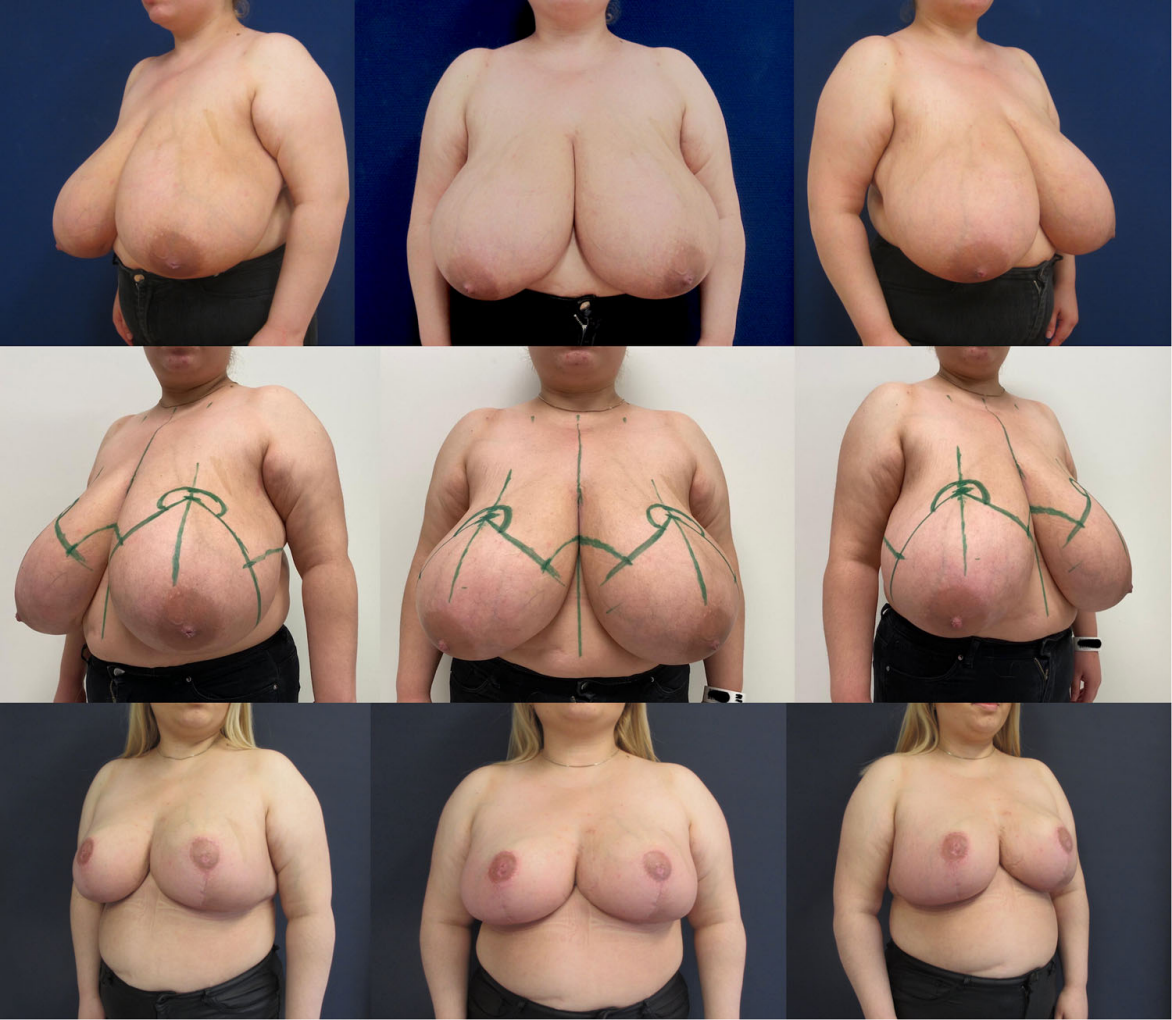
**Patient example 2**. 31-year-old patient with cup size 80 M, SN-NAC distance 39 cm right side and 38 cm left side, ptosis grade III by Regnault, BMI 32 kg/m^2^. Preoperative status (*above*), preoperative markings (*middle*), and 6 months postoperative (*below*) after *Double-Unit Superomedio-Central *(*DUS*)* Pedicled* Inverted-T Reduction Mammaplasty, form stable breast shape and good upper pole projection. Resection weight *right side* **2124 g** and *left side* **2248 g** (BMI: body mass index kg/m^2^; SN: sternal notch, NAC: sternal notch–nipple–areolar complex)

**Fig. 9:**
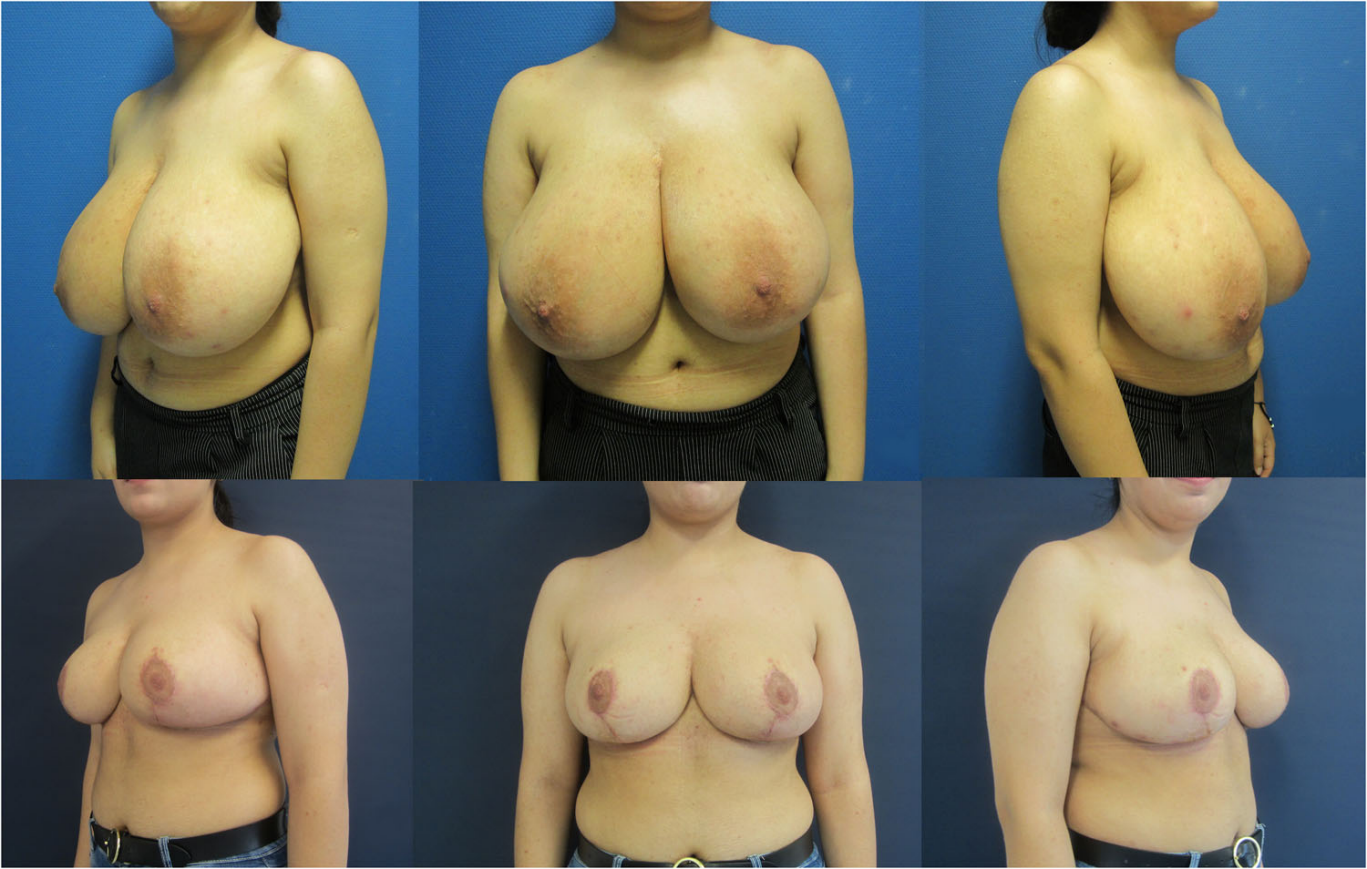
**Patient example 3**. 20-year-old patient with cup size 75 G, SN-NAC distance 36 cm right side and 34 cm left side, ptosis grade II by Regnault, BMI 26 kg/m^2^. Preoperative status (*above*), and 12 months postop (*below*) after *Double-Unit Superomedio-Central *(*DUS*)* Pedicled* Inverted-T Reduction Mammaplasty, form stable breast shape and good upper pole projection. Resection weight *right side* **1602 g** and *left side* **1150 g**. (BMI: body mass index kg/m^2^; SN: sternal notch, NAC: nipple–areolar complex)

**Fig. 10:**
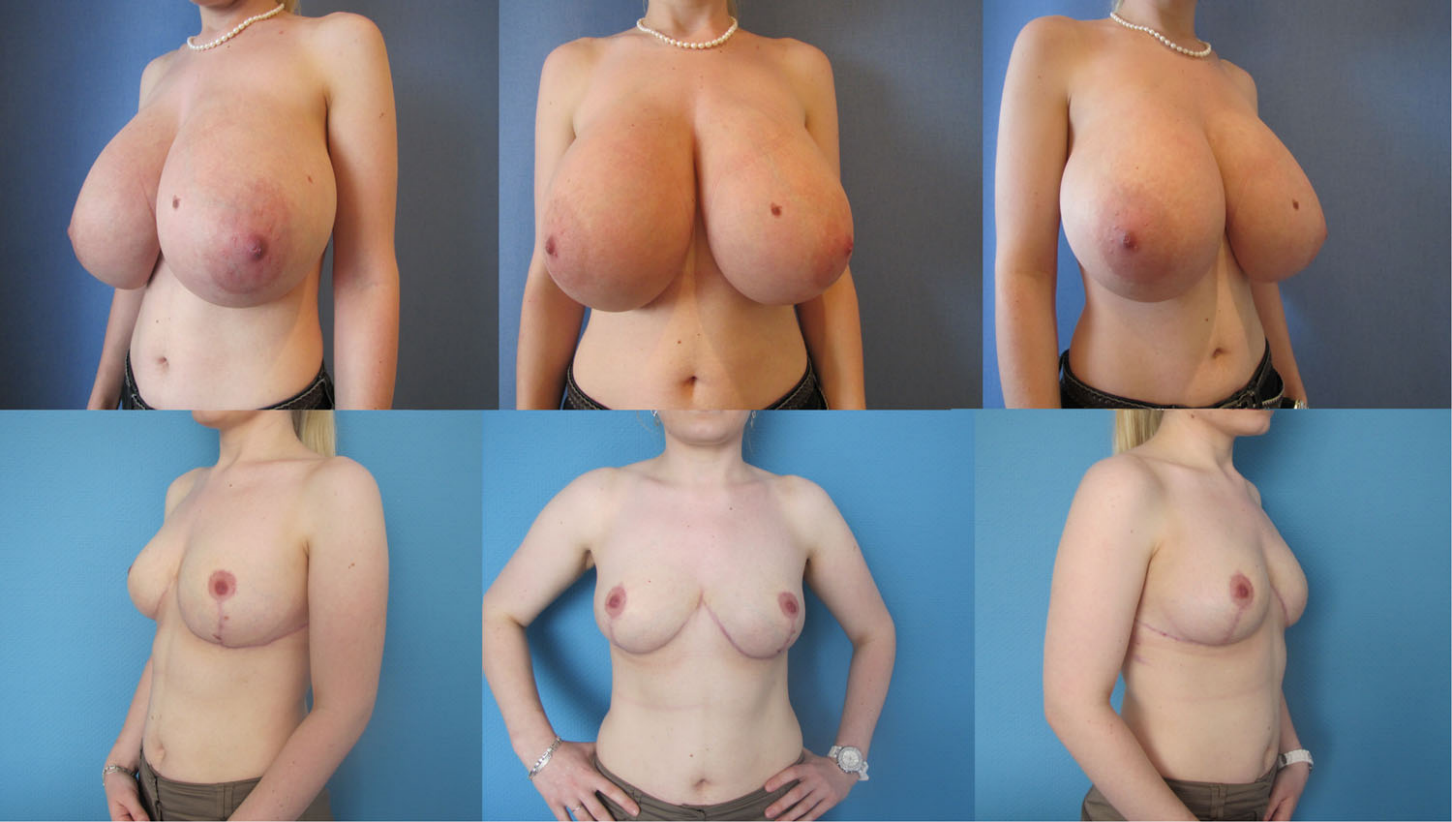
**Patient example 4**. 23-year-old patient with cup size 75 K, SN-NAC distance 34 cm right side and 34 cm left side, ptosis grade II by Regnault, BMI 24 kg/m^2^. Preoperative status (*above*), and 18 months postop (*below*) after *Double-Unit Superomedio-Central *(*DUS*)* Pedicled* Inverted-*T* Reduction Mammaplasty, form stable breast shape and good upper pole projection. Resection weight *right side* **1850 g** and *left side* **1800 g**. (BMI: body mass index kg/m^2^; SN: sternal notch, NAC: nipple–areolar complex)

**Fig. 11:**
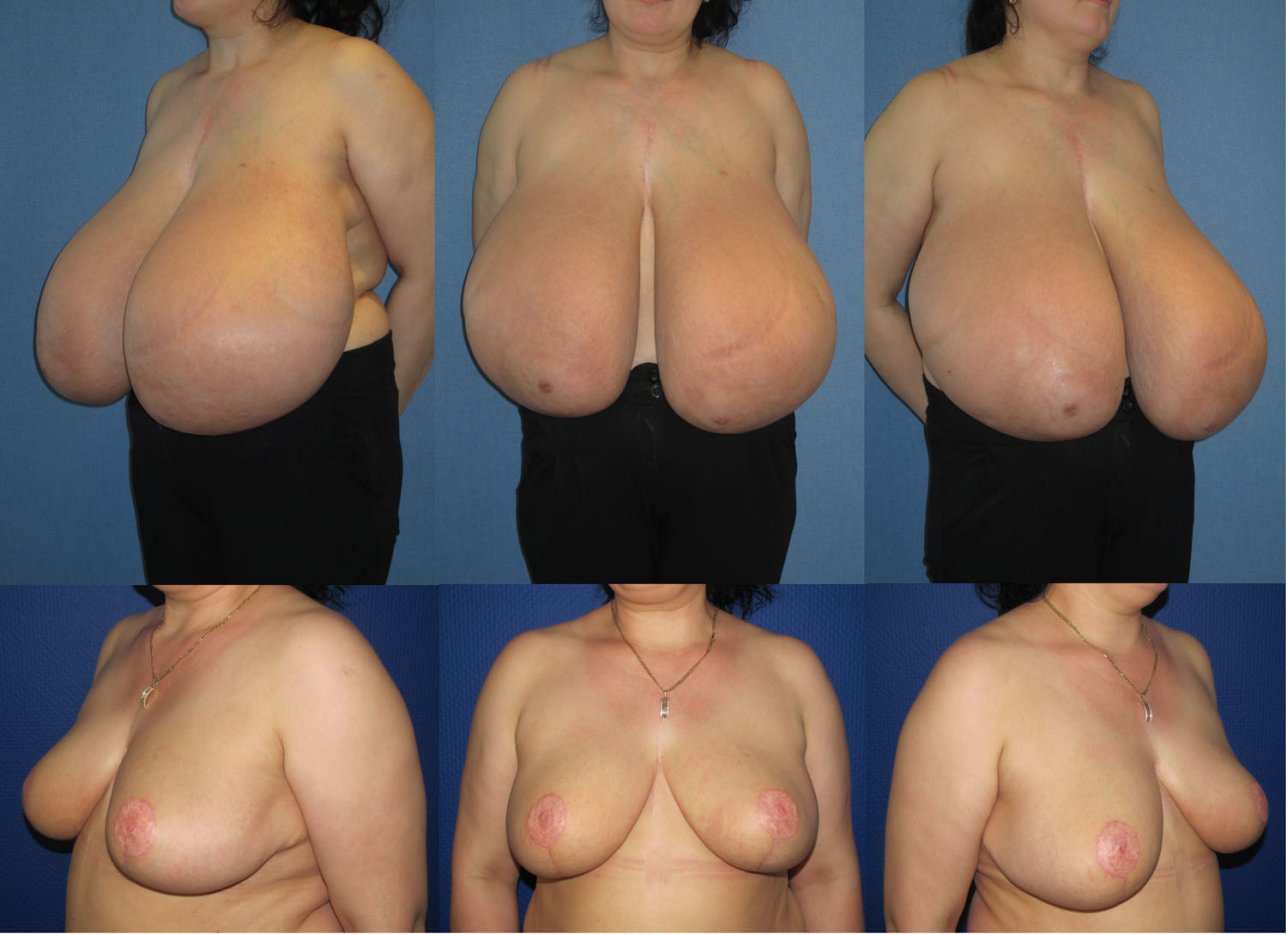
**Patient example 5**. 46-year-old patient with cup size 85 N, SN-NAC distance 54 cm right side and 57 cm left side, ptosis grade III by Regnault, BMI 29 kg/m^2^. Preoperative status (above), and 24 months postoperative (*below*) after free NAC graft and *Double-Unit Superomedio-Central *(*DUS*)* Pseudopedicled* Inverted-*T* Reduction Mammaplasty, form stable breast shape, good upper pole projection and mild NAC hypopigmentation. Resection weight *right side* **4200 g** and *left side* **4600 g**. (BMI: body mass index kg/m^2^; SN: sternal notch, NAC: nipple–areolar complex)

Moreover the multiplanar pillar sutures, placed to fix the gland vertically and in the *T*-junction, are used to decrease tension on the scars and to obtain a long-term breast shape by increasing the breast projection and reducing the risk of a bottoming out in the further course. *T* junction breakdown is a frequently occurring problem with rates up to 18% [4, 39]. In our collective we could reduce this minor complication to two breasts (3.6%) by commencement of the key sutures starting laterally so that the lateral skin excess is pushed medially to relieve tension at the *tripod point* and by placing multiplanar pillar sutures including an anchor suture in the *T* junction to avoid any stitch-out in this very vulnerable region (Fig. 4D). However, patients need to be aware that delayed healing is not an uncommon problem in gigantomastia. Immediate and late complication rates in gigantomastia can be found in literature up to 36% [27]. The overall complication rate in this series remained generally low (14.5%) (in comparison with literature regarding reduction mammaplasty in case of gigantomastia, Table 4), with one acute hematoma requiring evacuation (1.8%), only one total NAC loss (1.8%) in a strong smoking patient and SN-NAC distance of 42 cm that could be reconstructed by a skate flap and areolar tattooing and two NAC epidermiolysises (3.6%) that healed by secondary intention. Free NAC grafts were solely performed in extreme SN-NAC distances > 45 cm; in our collective in four breasts (see also patient examples Figs. 8, 9, 10, 11). In addition, NAC grafting is associated with loss of NAC sensation, lack of nipple projection, nipple hypopigmentation, and loss of lactation. Thus, it should not be performed on women of childbearing age who plan to breastfeed or women who want to preserve nipple sensation and erection.

**Table 4:** Relevant chronologically listed publications regarding gigantomastia—Comparison of techniques/pedicles, results, complications and outcome parameters

Authors (Year)	Technique/Pedicle	No. of breasts	Mean resection weight	Overall complications	Acute hematoma	Complete NAC loss	Secondary revisions	NAC sensibility	Aesthetic satisfaction
Nahabedian et al. [40]	]*Medial *pedicle	45	right 1580 g, left 1627 g	0%	0%	0%	0%	98% retained	97% satisfied
Misirlioglu et al. [41]	Vertical with *free* *NAC* *graft* (two cases)	2	1^st^ case 4100 g, 2^nd^ case 4100 g	0%	0%	0%	50%; 1 dog ear	NA	both satisfied
Lacerna et al. [42]	*Inferior* pedicle	30	range from 2000–4000 g	6.7%	0%	0%	NA	NA	NA
Azzam et al. [10]	Vertical with *superior* pedicle	53 >1000 g	right 807.9 g, left 822.4 g	36%	0%	3.8% gland. necrosis	24.5% scar and volume revision	NA	NA
Landau et al. [4]	*Superomedial* pedicle	122	right 1360 g, left 1398 g	24.6%	0%	0%	3.8%	NA	NA
Roehl et al. [43]	*Inferior *pedicle and *free NAC graft*	170 >1000 g	NA	50%	4%	1%	NA	NA	NA
Mojallal et al. [2]	*Posterosuperior* pedicle	100	1231 g	8%	0%	0%	0%	NA	74% “very good”, 18% “good”
Amini et al. [44]	Vertical with *superomedial* pedicle	46	right 1303 g, left 1245 g	21.7%	2.2%	0%	4.3%, 2 dog ears	1 “hyposensibility”	91% “very satisfied”
Lugo et al. [3]	*Superomedial *pedicle	400	right 1277 g, left 1283 g	10. 5%	NA	0%	NA	98% reported NAC sensation	NA
Hammond et al. [32]	Short-Scar Periareolar *Inferior* pedicle	140	1336 g	37%	0%	0%	5.7%	NA	NA
Karacor-Altuntas et al. [7]	*Central *pedicle without free nipple	434	right 1496 g, left 1417 g	4.1%	0%	0%	NA	100% good NAC sensation	NA
Karacaoglu et al. [8]	*Septum-Inferior-Medial* (SIM)-based pedicle	52	1512 g	8.3%	0%	0%	NA	intact in all cases	81% very satisfied
Ulusal et al. [45]	*Superior* pedicle	80	3066 ± 944 g	6.25%	1.3%	2.5%	17.5%	NA	Mean score 4,65 (0–5)
Kemaloǧlu et al. [9]	*Inferior *vs. *Superomedial* pedicle	50 vs. 50	right 1320 g, left 1350 g vs. right 1380 g, left 1310 g	16% vs. 8%	0%	0%	NA	4% decreased sensation vs. 4% decreased sensation	NA
Elmelegy et al. [46]	*Medial-Lateral* Bipedicle inverted T	72 > 1000 g	1433 g	6.9%	NA	1.3%	NA	1 “hypo-sensibility”	NA
Wolter et al. [47]	*Superomedial pedicle*	294	right 1413, left 1366 g	7.8%	0.3%	3.1%	16% NAC, scar and contour revisions	88% “very good” and “good”	93% “very satisfied” and “satisfied”

(*NAC* nipple-areola-complex, *NA* not available)

Although the Superomedio-Central Pedicle allows to support the NAC’s blood supply even in very elongated SN-NAC distances, we recommend that this decision should be supplemented by intraoperatively checking signs of venous congestion concerning the vascular NAC supply. If there is any suspicious NAC perfusion regarding a venous congestion, a free NAC graft should be performed. In 1922, Thorek was the first who described a free-nipple reduction mammaplasty in gigantomastia cases [48]. Recent publications still recommend a free NAC graft in severe gigantomastia cases [16, 41]. We agree that free NAC grafting should generally be reserved for high-risk, older patients, when shorter operating times are paramount. Only a minority of publications analysed the results regarding NAC sensibility, secondary revisions and satisfaction with the aesthetic result. It is concluded that NAC sensibility was preserved by securing the robust and major neurovascular supply by the Superomedio-Central pedicle (83% rated subjectively the sensibility as “high” and “medium”). The symmetry achieved with this method and consecutively the overall aesthetic outcome was rated by the patients very high (91% were “very satisfied” and “satisfied”).

## Conclusion

The *Double-Unit Superomedio-Central (DUS) pedicled* inverted-*T* incision for reduction mammaplasty in gigantomastia is a reproducible and versatile technique. The preservation of the septum-based anterior intercostal artery perforators enhances the reliability of the neurovascular supply to the  nipple-areolar complex. This modified procedure is very effective to achieve volume reduction and aesthetically pleasing reproducible results with a low complication rate in gigantomastic cases.

## Supplementary Information

Below is the link to the electronic supplementary material.

Supplementary file1 (MP4 819508 KB)

Supplementary file2 (MP4 336967 KB)
